# iDTI-ESBoost: Identification of Drug Target Interaction Using Evolutionary and Structural Features with Boosting

**DOI:** 10.1038/s41598-017-18025-2

**Published:** 2017-12-18

**Authors:** Farshid Rayhan, Sajid Ahmed, Swakkhar Shatabda, Dewan Md Farid, Zaynab Mousavian, Abdollah Dehzangi, M. Sohel Rahman

**Affiliations:** 1 Department of Computuer Science and Engineering, United International University, House 80, Road 8A, Dhanmondi, Dhaka 1209 Bangladesh; 2 Department of Computer Science, School of Mathematics, Statistics, and Computer Science, University of Tehran, Tehran, Iran; 3 Department of Computer Science, Morgan State University, Baltimore, Maryland USA; 4 Department of Computer Science and Engineering, Bangladesh University of Engineering and Technology, ECE Building, West Palashi, Dhaka 1205 Bangladesh

## Abstract

Prediction of new drug-target interactions is critically important as it can lead the researchers to find new uses for old drugs and to disclose their therapeutic profiles or side effects. However, experimental prediction of drug-target interactions is expensive and time-consuming. As a result, computational methods for predictioning new drug-target interactions have gained a tremendous interest in recent times. Here we present iDTI-ESBoost, a prediction model for identification of drug-target interactions using evolutionary and structural features. Our proposed method uses a novel data balancing and boosting technique to predict drug-target interaction. On four benchmark datasets taken from a gold standard data, iDTI-ESBoost outperforms the state-of-the-art methods in terms of area under receiver operating characteristic (auROC) curve. iDTI-ESBoost also outperforms the latest and the best-performing method found in the literature in terms of area under precision recall (auPR) curve. This is significant as auPR curves are argued as suitable metric for comparison for imbalanced datasets similar to the one studied here. Our reported results show the effectiveness of the classifier, balancing methods and the novel features incorporated in iDTI-ESBoost. iDTI-ESBoost is a novel prediction method that has for the first time exploited the structural features along with the evolutionary features to predict drug-protein interactions. We believe the excellent performance of iDTI-ESBoost both in terms of auROC and auPR would motivate the researchers and practitioners to use it to predict drug-target interactions. To facilitate that, iDTI-ESBoost is implemented and made publicly available at: http://farshidrayhan.pythonanywhere.com/iDTI-ESBoost/.

## Introduction

Targeted drug design is one of the key techniques in therapeutic drug discovery^1^. Prediction of new drug target interactions can help researchers to find new uses for old drugs and to discover their therapeutic profiles or side effects^2–4^. Since experimental prediction of drug-target interaction is expensive and time-consuming, computational methods have been gaining increasing popularity in recent years^5,6^.

During the past two decades, a wide range of computational approaches such as ligand-based methods^7,8^, target or receptor based methods^9,10^, gene ontology based methods^11^, literature text mining methods^12,13^ have been proposed to address the drug-target interaction. The performance and effectiveness of the ligand-based methods degrade due to the decrease in the the number of known ligands of a target protein. Receptor based methods often use docking simulation^14^ and heavily rely on the available three dimensional native structure of the protein targets. It is important to note that finding three-dimensional structures of the proteins is by it self is a costly and time-consuming task which is done using experimental methods such as NMR and X-ray Crystallography. Moreover, three dimensional structures are very difficult to predict for ion channel proteins and G-protein coupled receptors (GPCRs). In addition, the tremendous growth in the Biomedical literature has increased the redundancy problem of the compound names or the gene names and has been the main obstacle for literature based systematic text mining methods.

Recently, chemo-genomic methods^15^ have been attempted to identify drug-target interactions. This type of methods are mainly based on machine learning^16,17^, graph theory^18,19^ and network methods^20,21^. In the literature of the supervised learning setting, several classification algorithms have been found to be applied for this task. Examples include support vector machine^22,23^, deep learning^24^, fuzzy logic^25^, and nearest neighbor^26^. Yamanishi *et al*.^16^ first proposed a mechanism to formalize the inference of the drug–target identification as a supervised learning problem. In that pioneering work, they also proposed a gold standard dataset that had been later used extensively in the literature^22,24,27^. In a subsequent work, the same authors^27^ explored the association among pharmacological space and chemical space with the network topology of drug-target interactions and applied distance-based learning. Wang *et al*. proposed RLS-KF^28^ that uses regularized least squares method integrated with nonlinear kernel fusion. Drug-based similarity inference (DBSI) was proposed in^21^ utilizing two dimensional chemical structural similarity. Another method, KBMF2K, was proposed in^29^ that used chemical and genomic kernels and bayesian matrix factorization. Later on other noteworthy methods such as NetCBP^30^, DASPfind^31^, SELF-BLM^23^ have been proposed to solve thi sproblem. Recently^22^ used bigram based features extracted from Position Specific Scoring Matrix (PSSM) as molecular fingerprint to tackle this problem.

Since the three dimensional native structure of most of the protein targets are not available, most of the supervised learning methods in the literature do not exploit the structure based features. Huang *et al*.^32^ used extremely randomized trees model and represented the proteins as pseudo substitution matrix generated from its amino acid sequence information and the drugs as moelcular fingerprint. In another recent work, Wang *et al*.^33^ explored PSSM based features and drug fingerprints and used rotation forest based predictor. Among other recent works are similarity based method used by Yuan *et al*.^34^, self organizing theory used by Duran *et al*.^35,36^ and ensemble method used by Ezzat *et al*.^37,38^. Recently, a comprehensive literature review on the computational methods in drug-target interaction prediction was conducted by Chen *et al*.^39^.

In this paper, we present iDTI-ESBoost, a method for **i**dentification of **D**rug **T**arget **I**nteraction Using **E**volutionary and **S**tructural Information with **Boost**ing. We exploit the structural features along with the evolutionary features to predict drug-protein interactions. Our work was inspired due to the modern successful secondary structural prediction tools like SPIDER2^40,41^ and its use to generate features in supervised learning and classification^42,43^. Our proposed method uses a novel set of features extracted using structural information along with the evolutionary features and molecular fingerprints of drugs. To handle the large amount of imbalance in the data, we propose a novel balancing method and use it along with a boosting algorithm. As a result, iDTI-ESBoost has shown to be superior due to its prediction results on a widely used gold standard data set compared to the other existing methods found in the literature. Our method is publicly available to use at: http://farshidrayhan.pythonanywhere.com/iDTI-ESBoost/.

The rest of the paper is organised as follows which is suggested in^44^: description of dataset, formulation of statistical samples, selection and development of a powerful classification algorithm, demonstration of the performance of the predictor using cross-validation, implementation of web server followed by a conclusion.

## Results and Discussion

In this section, we present the results of our experiments. All the methods were implemented in Python language using Python3.4 version and Scikit-learn library^45^ of Python was used for the implementation of the machine learning algorithms. All experiments were conducted on a Computing Machine hosted by CITS, United International University. Also, each of the experiments was carried out 5 times and the average of the results is reported. We perform several types of experiments. In particular, we conduct four different sets of experiments as follows. First we investigate effectiveness and applicability of the different feature groups as mentioned in Table 1. Note that, in Table 1, four different feature groups, namely, A, B, C and D, were formed. Secondly, we investigate the effectiveness of the classifiers used in our research. Subsequently, we investigate the effectiveness of the balancing methods applied on our highly imbalanced datasets. Finally, we compare iDTI-ESBoost against the state-of-the-art methods found in the literature.

**Table 1:** Summary of evolutionary and structural features used for protein targets and fingerprint features for drugs.

Feature Group	Number of Features	Feature Type	Group
Molecular finger print	881	drug	
PSSM bigram	400	target	A
Secondary Structure Composition	3	target	B
Accessible Surface Area Composition	1		
Torsional Angles Composition	8		
Torsional Angles Auto-Covariance	80	target	C
Structural Probabilities Auto-Covariance	30		
Torsional Angles bigram	64	target	D
Structural Probabilities bigram	9		
Total	1476		

The “Group” column shows different feature groups used in our experiments and will be discussed in a later section.

### Effectiveness of Feature Groups

We created four different feature groups to see the effects of the different sets of features on the classifier performance. The feature groups have already been reported in Table 1. Group A contains 1281 features and was previously used in^22^. We further added other groups, namely, B, C and D, incrementally in that order with the base feature group i.e., Group A and achieve features of size 1293, 1403 and 1476, respectively. We have performed two sets of experiments to test the effectiveness of the feature groups. In both of these experiments we changed the feature groups and used different classifiers and applied different balancing methods on the data to analyze the effect. Results of these experiments are reported respectively in Tables 2 and 3.

**Table 2:** Performance of AdaBoost, Random Forest and Support Vector Machine classifiers on the gold standard datasets in terms of area under Receiver Operating Characteristic (ROC) curve (auROC) and area under precision recall curve (auPR) using different feature group combinations and random under sampling.

Dataset	Feature Combination	Classifier	auPR	auROC
enzymes	A	AdaBoost	0.54	0.9530
		Random Forest	0.43	0.9457
		SVM	**0**.**64**	**0**.**9647**
	A, B	AdaBoost	**0**.**51**	0.9431
		Random Forest	0.49	0.9445
		SVM	0.48	**0**.**9502**
	A, B, C	AdaBoost	**0**.**66**	**0**.**9638**
		Random Forest	0.48	0.9334
		SVM	0.41	0.9360
	A, B, C, D	AdaBoost	**0**.**65**	**0**.**9689**
		Random Forest	0.50	0.9493
		SVM	0.63	0.9628
ion channels	A	AdaBoost	**0**.**36**	0.9271
		Random Forest	0.33	0.9232
		SVM	0.25	**0**.**9467**
	A, B	AdaBoost	**0**.**33**	0.9191
		Random Forest	0.30	0.8898
		SVM	0.23	**0**.**9213**
	A, B, C	AdaBoost	**0**.**34**	0.9202
		Random Forest	0.31	0.8734
		SVM	0.23	**0**.**9213**
	A, B, C, D	AdaBoost	**0**.**43**	**0**.**9369**
		Random Forest	0.40	0.9234
		SVM	0.14	0.6723
GPCRs	A	AdaBoost	**0**.**29**	**0**.**8856**
		Random Forest	0.23	0.8743
		SVM	0.18	0.7832
	A, B	AdaBoost	**0**.**29**	**0**.**8834**
		Random Forest	0.22	0.8698
		SVM	0.15	0.7802
	A, B, C	AdaBoost	**0**.**35**	**0**.**9116**
		Random Forest	0.31	0.9034
		SVM	0.15	0.7945
	A, B, C, D	AdaBoost	**0**.**31**	**0**.**9128**
		Random Forest	0.30	0.9168
		SVM	0.21	0.7896
nuclear receptors	A	AdaBoost	**0**.**41**	**0**.**8145**
		Random Forest	0.23	0.7519
		SVM	0.19	0.7898
	A, B	AdaBoost	**0**.**43**	**0**.**7969**
		Random Forest	0.29	0.7723
		SVM	0.20	0.6789
	A, B, C	AdaBoost	**0**.**36**	**0**.**7590**
		Random Forest	0.21	0.7234
		SVM	0.21	0.6971
	A, B, C, D	AdaBoost	**0**.**33**	**0**.**7946**
		Random Forest	0.29	0.7145
		SVM	0.20	0.7287

**Table 3:** Performance of Adaboost classifier on different datasets in terms of area under Receiver Operating Characteristic (ROC) curve (auROC) and area under precision recall curve (auPR) using different feature group combinations and balancing methods.

Dataset	Feature Combination	Balancing Method	auPR	auROC
enzymes	A	random	0.54	0.9530
		clustered	0.58	0.9493
	A, B	random	0.51	0.9431
		clustered	0.59	0.9353
	A, B, C	random	0.66	0.9638
		clustered	0.63	0.9577
	A, B, C, D	random	0.65	**0**.**9689**
		clustered	**0**.**68**	0.9598
ion channels	A	random	0.36	0.9271
		clustered	0.38	0.8982
	A, B	random	0.33	0.9191
		clustered	0.41	0.8902
	A, B, C	random	0.34	0.9202
		clustered	0.45	0.9021
	A, B, C, D	random	0.43	**0**.**9369**
		clustered	**0**.**48**	0.9051
GPCRs	A	random	0.29	0.8856
		clustered	0.48	0.9189
	A, B	random	0.29	0.8834
		clustered	0.49	0.8968
	A, B, C	random	0.35	0.9116
		clustered	**0**.**50**	0.8890
	A, B, C, D	random	0.31	0.9128
		clustered	0.48	**0**.**9322**
nuclear receptors	A	random	0.41	0.8145
		clustered	0.79	0.9270
	A, B	random	0.43	0.7969
		clustered	0.32	0.8715
	A, B, C	random	0.36	0.7590
		clustered	0.57	0.8935
	A, B, C, D	random	0.33	0.7946
		clustered	**0**.**79**	**0**.**9285**

Table 2 reports the performance of three different classifiers on the four datasets during our experiments. In this step, we have produced the results for different combination of feature groups by adding them in a forward selection scheme by sorting them based on their individual performance for Nuclear Receptors. For the Nuclear Receptors, the best results achieved using Feature group A, and followed by B, C, and D, consequently. We used the individual performance for Nuclear Receptors as it produced the most distinguished performance for different input feature group. Therefore, we first evaluate the performance using feature group A, then added B, C, and finally D, sequentially. We have produced the results for each feature group as well as different combination of these feature gorups based on sequential forward selection. These results are available and provided as a supplementary material (Supplementary File 1).

Note that, though this experiment was intended for classifier selection, we clearly see that the best results in terms of auPR and auROC were found only when the structural features were added. For enzymes dataset, the best result in terms of auPR was 0.66 found with the combination A, B, C which is using structural composition and structural auto-covariance groups with PSSM-bigram and molecular fingerprint based features. This result is slightly better than the case when we use all the features A, B, C, D and get auPR of 0.66. In terms of auROC, the results are somewhat comparable to each other; however, the best result is achieved when all the four feature groups are used together. Thus enzyme dataset shows the effectiveness of structural information based features. Using iDTI-ESBoost for ion channels and GPCRs datasets we attain similar performance in terms of auPR. However it achieved highest auPR value when only the composition features (Group B) were added with the base features for Nuclear receptors dataset. The increase in the value of auROC clearly reveals the effectiveness of the structural features (Groups B, C, D) when added to the base feature (Group A).

As it is shown in Table 2, due to differences in the explored problems in this study, the results for different combination of feature groups, and different classifiers are not fully consistent. However, The aim of this experiment is to compare and analyse the performance of our method with respect to different combination of feature groups and different problems and find the best combination for that given specific problem. By conducting this comprehensive comparison, we are able to find the best classifier among those that we investigated here with respect to the combination of feature groups that we studied for a given problem.

The next set of experiments were run to show the performance of different balancing or under sampling methods in the training data using various feature groups. These results are shown in Table 3. These experiments were run using the AdaBoost classifier. The results in Table 3 clearly shows that for all the datasets, the best results in terms of auPR and auROC were found when structural features have been added. In case of the GPCRs, the auPR was found to be the highest at 0.5 when three feature groups, namely, Groups A, B, and C have been combined. Apart from this, in all other datasets, the combination of all four groups has shown superior performance both in terms of auPR and auROC. Our hypothesis that the added structural features play a significantly important role in the prediction of drug-target interaction is thus justified according to these experiments.

### Effectiveness of the AdaBoost Classifier

**Figure 1:**
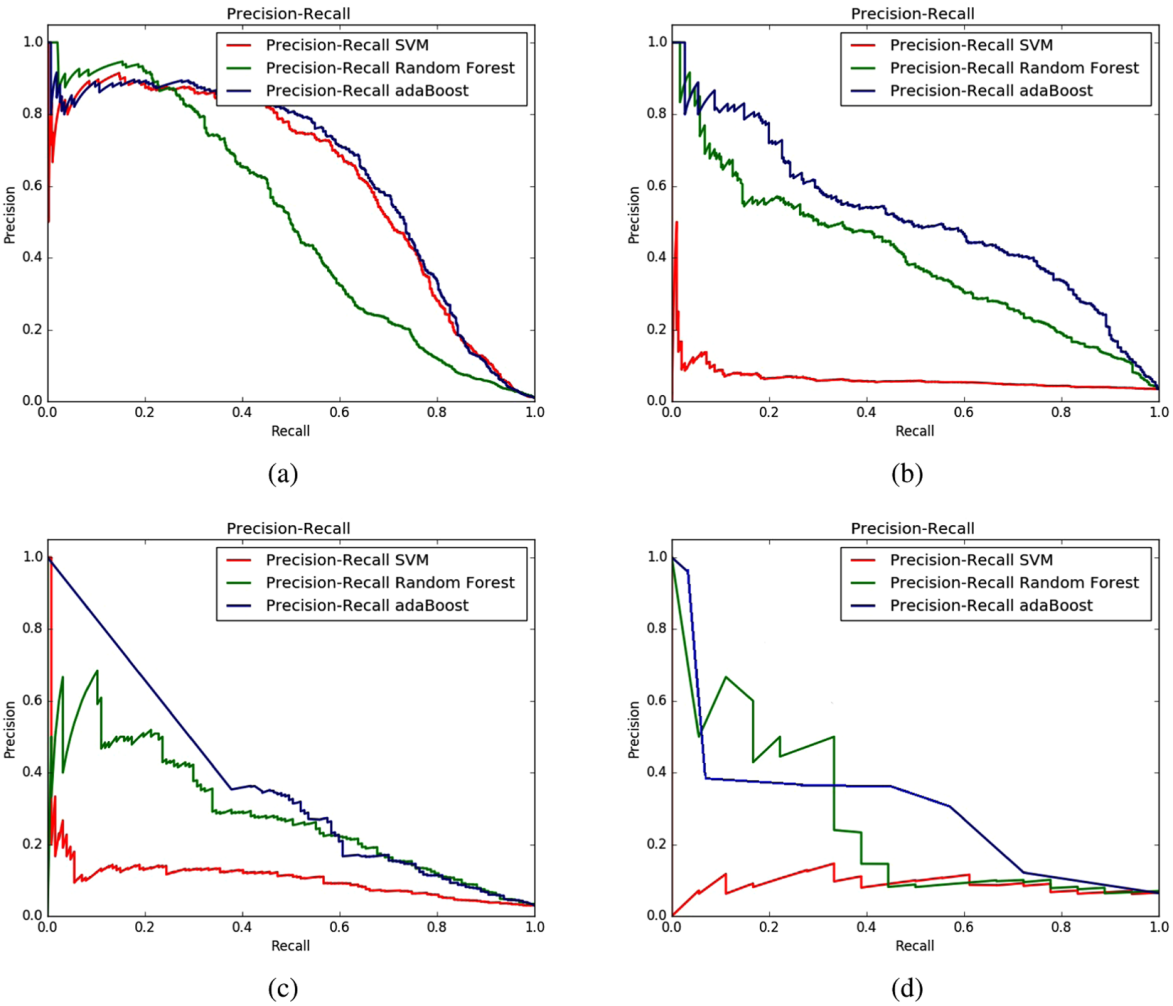
Precision-Recall curves of different classifier algorithms using random under sampling and all the feature combinations on four datasets: (**a**) enzymes (**b**) ion channels (ic) (**c**) GPCRs (**d**) nuclear receptors (nr).

To test and select the suitable classifier for our problem, we test three different classifiers namely, AdaBoost ensemble classifier^46^ with decision tree as its weak learner, Random Forest^47^ and Support Vector Machines (SVM)^48^. For these experiments, we used random under sampling as the balancing method. As features, four different combinations were used as has been mentioned already. The results in terms of auPR and auROC are presented in Table 2. Here for each of the datasets and feature groups combinations bold faced values in the table represents the highest values achieved for that combination. It is evident that except for one case in the enzymes dataset, AdaBoost classifier has shown superior performance in terms of auPR across all feature groups combinations. It is also worth-noting that for all datasets, the highest auPR value was achieved by AdaBoost. The precision-recall curves for these experiments across all feature groups combinations are illustrated in Fig. 1.

**Figure 2:**
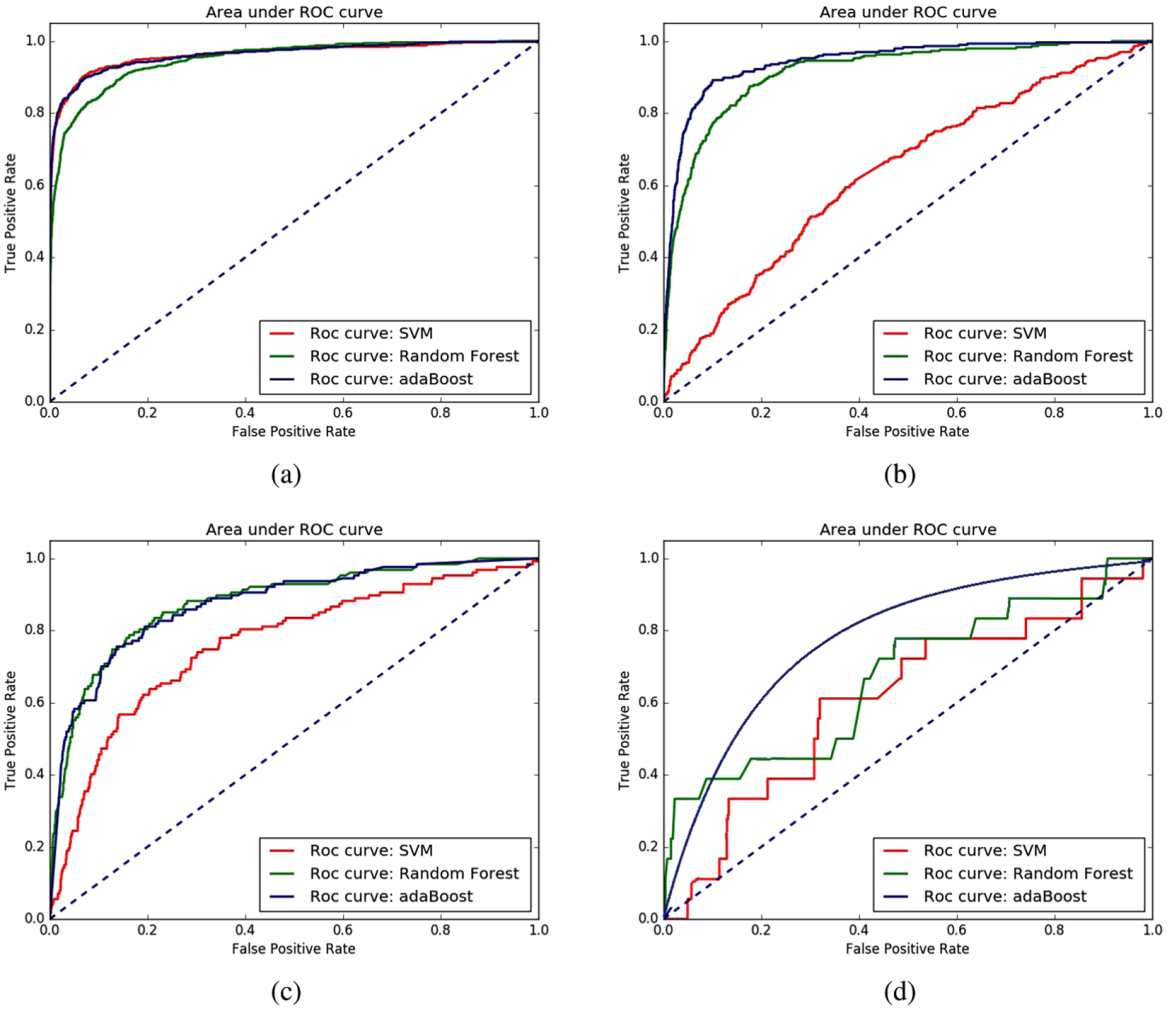
Receiver operating characteristic (ROC) curves of different classifier algorithms using random under sampling and all the feature combinations on four datasets: (**a**) enzymes (**b**) ion channels (ic) (**c**) GPCRs (**d**) nuclear receptors (nr).

In case of the ROC curves, achieved results are also in support of the selection of AdaBoost as a classifier. AdaBoost provides the highest auROC values for all the four datasets and it gives better auROC values for 11 out of 16 dataset-feature groups combinations. In other cases, SVM has achieved the highest auROC values, but only marginally so. The ROC curves for different classifiers across all feature groups combinations are illustrated in Fig. 2.

Considering the values of auPR and auROC curves on different datasets as shown in Table 2 and illustrated through the curves in Figs 1 and 2, we select AdaBoost as the classifier for iDTI-ESBoost. Note that, because of the huge imbalance in the datasets, with positive samples being much lower than the negative ones, the auPR curve is more important compared to the auROC curve and AdaBoost clearly outperforms the other two classifiers in terms of auPR values.

### Effectiveness of the Balancing Methods

The next set of experiments were run to test the effectiveness of the two different sampling methods on the datasets. The parameters used with AdaBoost classifier for random and cluster based under sampling are reported in Table 4.

**Table 4:** Parameters of AdaBoost Algorithm used with decision tree as weak classifier along with different balancing methods on four datasets.

Balancing method	Dataset	Max depth	Min sample split	Min samples Leaf	Criterion
random	enzymes	100	16	1	Gini impurity
	ion channels	8	4	1	Gini impurity
	GPCRs	6	3	1	Gini impurity
	nuclear receptors	5	7	2	Gini impurity
clustered	enzymes	110	1	1	Gini impurity
	ion channels	9	2	1	Gini impurity
	GPCRs	6	3	1	Gini impurity
	nuclear receptors	150	2	1	Gini impurity

**Figure 3:**
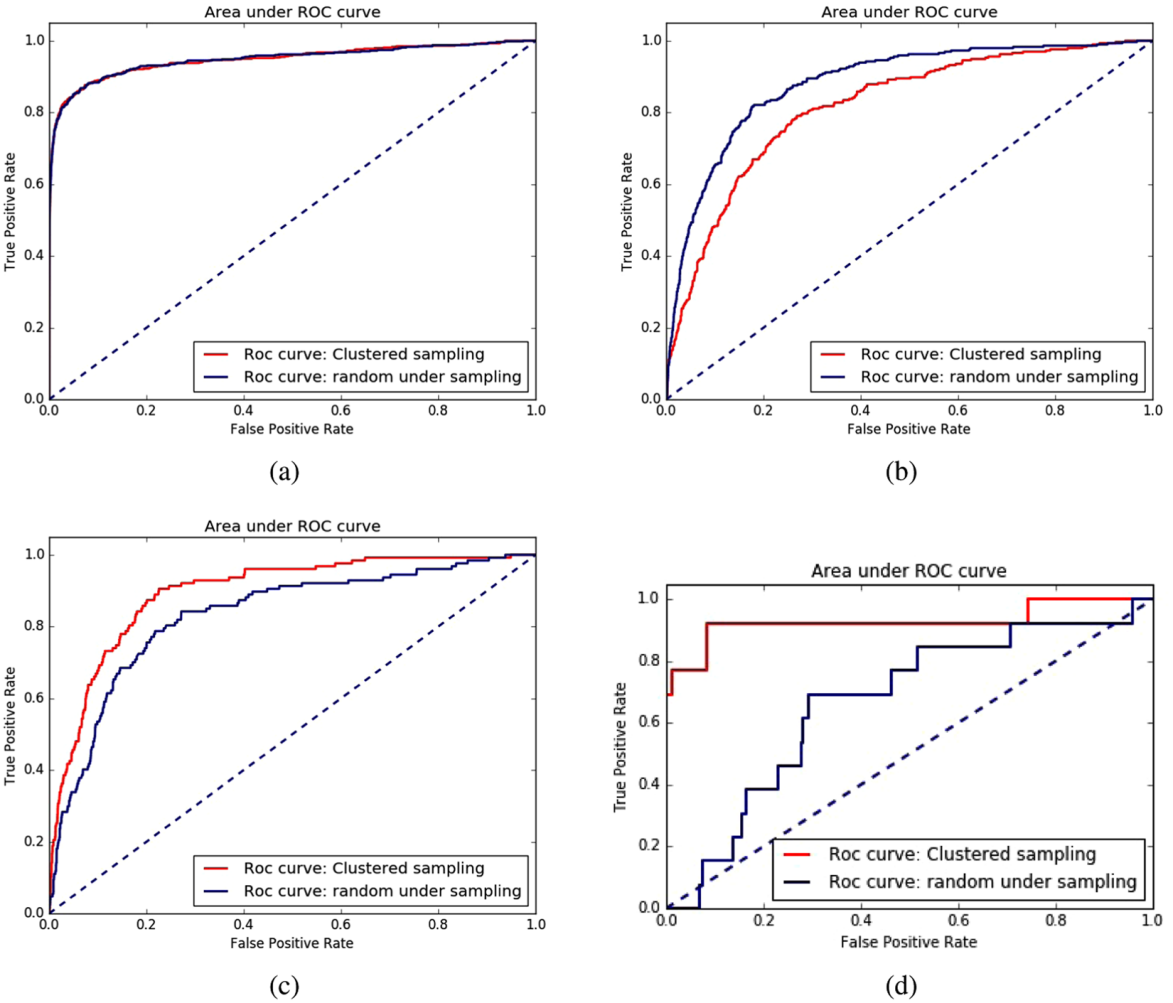
Receiver operating characteristic (ROC) curves of AdaBoost classifier showing differences between random under sampling and cluster based sampling using all the feature combinations on four datasets: (**a**) enzymes (**b**) ion channels (ic) (**c**) GPCRs (**d**) nuclear receptors (nr).

**Figure 4:**
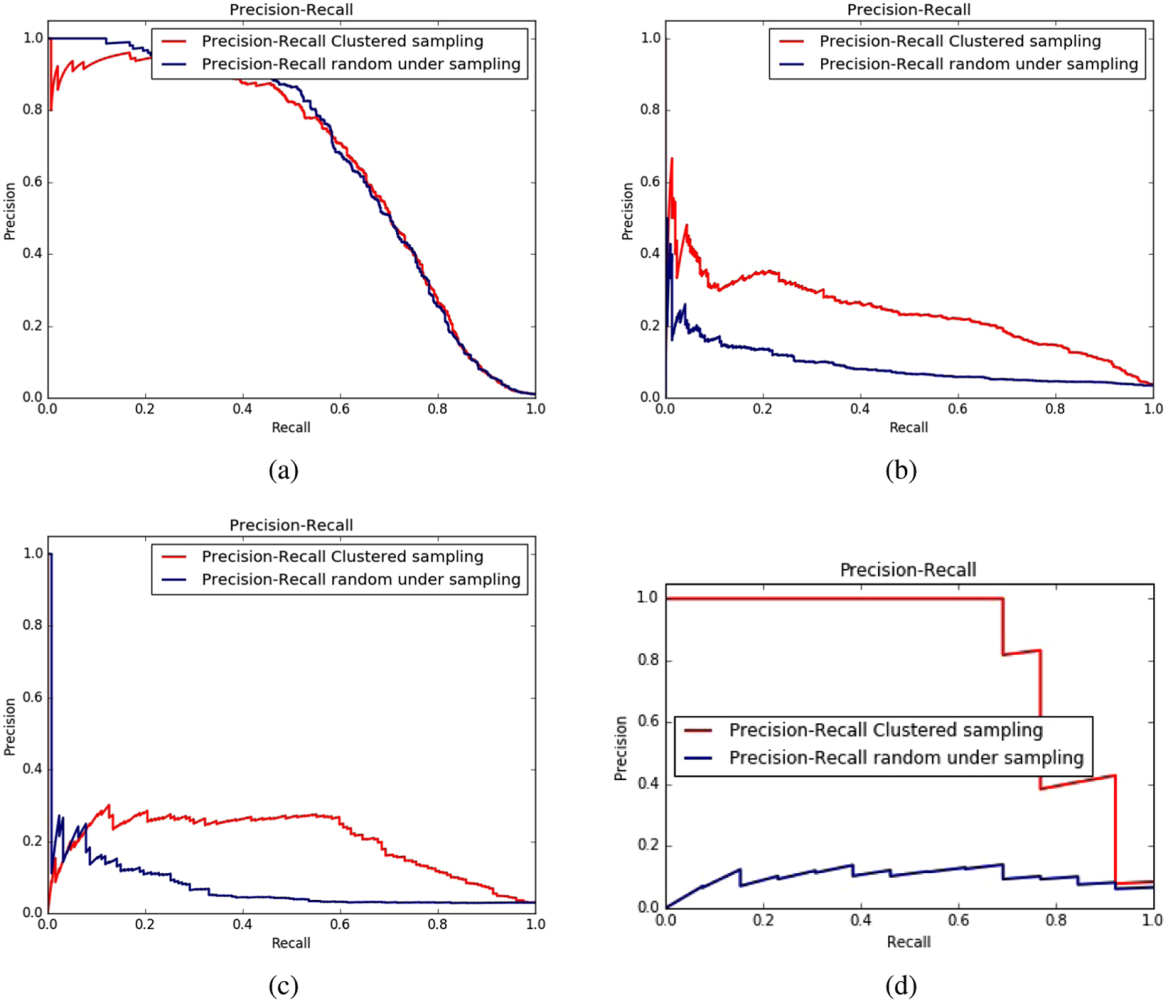
Precision vs Recall curves of AdaBoost classifier showing differences between random under sampling and cluster based sampling using all the feature combinations on four datasets: (**a**) enzymes (**b**) ion channels (ic) (**c**) GPCRs (**d**) nuclear receptors (nr).

For each of the datasets, we used four feature group combinations and used random and cluster based under sampling and report auPR and auROC values from cross-validation experiments in Table 3. We also show the ROC curves and auPR curves for all four datasets using all the features in Figs 3 and 4.

From the results reported in Table 3, it is worth-mentioning that in terms of auPR for all four datasets, cluster based sampling significantly outperforms random under sampling method. However, in terms of auROC curve, random sampling is slightly better than cluster based sampling in enzymes and ion channel datasets but the situation is in favor of cluster based sampling in GPCRs and nuclear receptors where it outperforms the random sampling method.

We have also analyzed the effect of the imbalance ratio of the four datasets with the different balancing methods used in this paper. Note that the performance of the random sampling and clustered sampling are similar in terms of auPR in the dataset enzymes with higher imbalance ratio. The performance drustically falls for random sampling for the nuclear receptor dataset which have the lowest imbalance ratio. In case of the other two datasets ion channels and GPCRs, though their imbalance ratio is similar the difference in the auPR resutls differ significantly for these two balancing methods. Thus we can not conclude any correlation of imbalance ratio with that of the performance of the balacning methods. Rather the number of instances seems to affect the performance of the balancing methods. Enzymes and ion channels datasets with larger number of samples seems to favor random sampling and relatively smaller datasets GPCRs and nuclear receptors produces best resutls when using clustered sampling.

### Comparison with Other Methods

Since the pioneering work of Yamanishi *et al*.^16^, many supervised learning methods have been applied to predict drug-target interactions on these standard benchmark gold standard datasets. However, a few of these methods^24,28^ do not use cross validation techniques and others^3,23^ do not use the same standard datasets. Our method uses molecular fingerprints and evolutionary and structural features for this supervised classification problem. Similar methods, albeit without utilizing the structural features and balancing techniques are reported in^22,49^. Most of the papers in the literature have used auROC curve as the main evaluation metric. We have compared the performance of our method on these four datasets with that of DBSI^21^, KBMF2K^29^, NetCBP^30^, Yamanishi *et al*.^16^, Yamanishi *et al*.^27^, Wang *et al*.^18^ and Mousavian *et al*.^22^ using auROC. The auROC values for all these methods along with iDTI-ESBoost are reported in Table 5.

**Table 5:** Performance of iDTI-ESBoost on the four benchmark gold datasets in terms on area under receiver operating characteristic curve (auROC) with comparison to other state-of-the-art methods.

Dataset	DBSI^21^	KBMF2K^29^	NetCBP^30^	Yamanishi *et al*.^16^	Yamanishi *et al*.^27^	Wang *et al*.^18^	Mousavian *et al*.^22^	iDTI-ESBoost
enzymes	0.8075	0.8320	0.8251	0.904	0.8920	0.8860	0.9480	**0**.**9689**
ion channels	0.8029	0.7990	0.8034	0.8510	0.8120	0.8930	0.8890	**0**.**9369**
GPCRs	0.8022	0.8570	0.8235	0.8990	0.8270	0.8730	0.8720	**0**.**9322**
nuclear receptors	0.7578	0.8240	0.8394	0.8430	0.8350	0.8240	0.8690	**0**.**9285**

From the values shown in bold in Table 5, we see that for all the datasets iDTI-ESBoost is able to significantly outperform all other previous state-of-the-art methods in terms of auROC. All the auROC values are greater than 90% which indicates the effectiveness of the classifier, balancing methods and the novel features proposed in this paper.

Moreover, in^22^ the authors argued in favor of auPR curve as a measure of evaluating the performance of classifiers for skewed datasets, especially in drug-target interaction where negative samples outnumber the positive samples. This argument does have merit as, logically, a mis-classification of positive samples or false negative should be more penalized in the score. To compare the performance in terms of auPR among iDTI-ESBoost with that in^22^, we reported the auPR values of the two predictors in Table 6. The results clearly shows that our method iDTI-ESBoost outperforms the predictor in^22^ in terms of auPR as well.

**Table 6:** Comparison of the performance of iDTI-ESBoost on the four benchmark gold datasets in terms on area under the precision-recall curve (auPR) with the state-of-the-art method in Mousavian *et al*.^22^.

Predictor	Enzymes	Ion channels	GPCRs	Nuclear receptors
Mousavian *et al*.^22^	0.546	0.390	0.282	0.411
iDTI-ESBoost	**0**.**680**	**0**.**480**	**0**.**500**	**0**.**790**

In Table 7, we report specificity, sensitivity, precision, MCC and F1-Score for four datasets using different feature group combinations as achieved by iDTI-ESBoost in experiments. Specificity and sensitivity are very high as reported in this table.

**Table 7:** Specificity, Sensitivity, Precision, MCC and F1 score for four datasets as achieved by iDTI-ESBoost using different feature groups.

Dataset	Feature Group	Specificity	Sensitivity	Precision	MCC	F1 score
enzymes	A	0.83	0.9	0.05	0.1962	0.10
	A, B	0.82	0.89	0.05	0.1812	0.09
	A, B, C	0.83	0.87	0.05	0.1762	0.09
	A, B, C, D	0.85	0.85	0.15	0.1889	0.10
Ion channels	A	0.78	0.81	0.13	0.2615	0.22
	A, B	0.78	0.84	0.14	0.256	0.24
	A, B, C	0.8	0.86	0.12	0.2980	0.20
	A, B, C, D	0.78	0.84	0.13	0.2913	0.20
GPCRs	A	0.78	0.84	0.12	0.254	0.20
	A, B	0.8	0.85	0.11	0.2760	0.20
	A, B, C	0.79	0.89	0.11	0.2797	0.19
	A, B, C, D	0.8	0.84	0.11	0.2647	0.19
Nuclear receptors	A	0.85	0.91	0.16	0.2141	0.27
	A, B	0.77	0.88	0.11	0.2154	0.19
	A, B, C	0.81	0.88	0.12	0.1798	0.20
	A, B, C, D	0.92	0.87	0.14	0.2253	0.24

### Predicting New Interactions

In addition to these, we have analyzed the results produced by the classification algorithm. From the false negatives predicted by iDTI-ESBoost, we noticed that there are a number of false negatives for which the prediction probability is very high for it to be considered as a negative sample. Similar approaches were adopted in^16,27^. In this paper, we suggest that the false negative interactions which are labeled as positive by our method with a very high prediction probability could be potential candidates for finding new positive interactions. A list of such interactions for four group of targets are given in Table 8. Ten interactions are reported for each of the datasets with highest prediction probability.

**Table 8:** New prediction made by iDTI-ESBoost for four gold standard datasets used in this paper.

Dataset	Protein Id	Drug Id	Drug Name	Score
Enzymes	hsa:10825	D00041	Threonine (USP)	0.7207
	hsa:4759	D00041	Threonine (USP)	0.7163
	hsa:129807	D00041	Threonine (USP)	0.7163
	hsa:4953	D00041	Threonine (USP)	0.7095
	hsa:1845	D00041	Threonine (USP)	0.7078
	hsa:9610	D00041	Threonine (USP)	0.7073
	hsa:6652	D00041	Threonine (USP)	0.7034
	hsa:1734	D00136	Haloperidol (JP17/USP/INN)	0.6995
	hsa:1178	D03643	Dalvastatin (USAN/INN)	0.6985
	hsa:8435	D03643	Dalvastatin (USAN/INN)	0.6962
Ion channels	hsa:285242	D00294	Diazoxide (JAN/USP/INN)	0.9407
	hsa:779	D00294	Diazoxide (JAN/USP/INN)	0.9366
	hsa:2561	D00294	Diazoxide (JAN/USP/INN)	0.9357
	hsa:785	D00294	Diazoxide (JAN/USP/INN)	0.9353
	hsa:11254	D00294	Diazoxide (JAN/USP/INN)	0.935
	hsa:3775	D00225	Alprazolam (JP17/USP/INN)	0.9339
	hsa:6263	D00294	Diazoxide (JAN/USP/INN)	0.932
	hsa:6324	D02261	Quinine hydrochloride hydrate (JP17)	0.9305
	hsa:6324	D02262	Quinine sulfate (USP)	0.9305
	hsa:6332	D02262	Quinine sulfate (USP)	0.8464
GPCRs	hsas:9052	D04625	Isoetharine (USP)	0.9311
	hsa:9052	D00632	Dobutamine hydrochloride (JP17/USP)	0.9311
	hsa:9052	D03880	Dobutamine lactobionate (USAN)	0.9311
	hsa:9052	D03881	Dobutamine tartrate (USP)	0.9311
	hsa:1909	D03621	Cyclizine (INN)	0.931
	hsa:57105	D01712	Theophylline sodium acetate (JAN)	0.9307
	hsa:155	D02671	Mesoridazine (USAN/INN)	0.9306
	hsa:148	D02614	Denopamine (JAN/INN)	0.9303
	hsa:155	D00480	Promethazine hydrochloride (JP17/USP)	0.9302
	hsa:1909	D00480	Promethazine hydrochloride (JP17/USP)	0.9302
Nuclear receptors	hsa:2099	D01132	Tazarotene (JAN/USAN/INN)	0.9792
	hsa:2101	D00956	Nandrolone phenpropionate (USP)	0.9755
	hsa:2101	D00443	Spironolactone (JP17/USP/INN)	0.9758
	hsa:2099	D00316	Etretinate (JAN/USAN/INN)	0.9602
	hsa:9971	D00316	Etretinate (JAN/USAN/INN)	0.9593
	hsa:2101	D00327	Fluoxymesterone (JP17/USP/INN)	0.9591
	hsa:2101	D00088	Hydrocortisone (JP17/USP/INN)	0.9571
	hsa:2101	D00075	Testosterone (JAN/USP)	0.9558
	hsa:2099	D00565	Fenofibrate (JAN/INN)	0.9557
	hsa:2101	D00462	Oxandrolone (JAN/USP/INN)	0.9557

### Web Server Implementation

**Figure 5:**
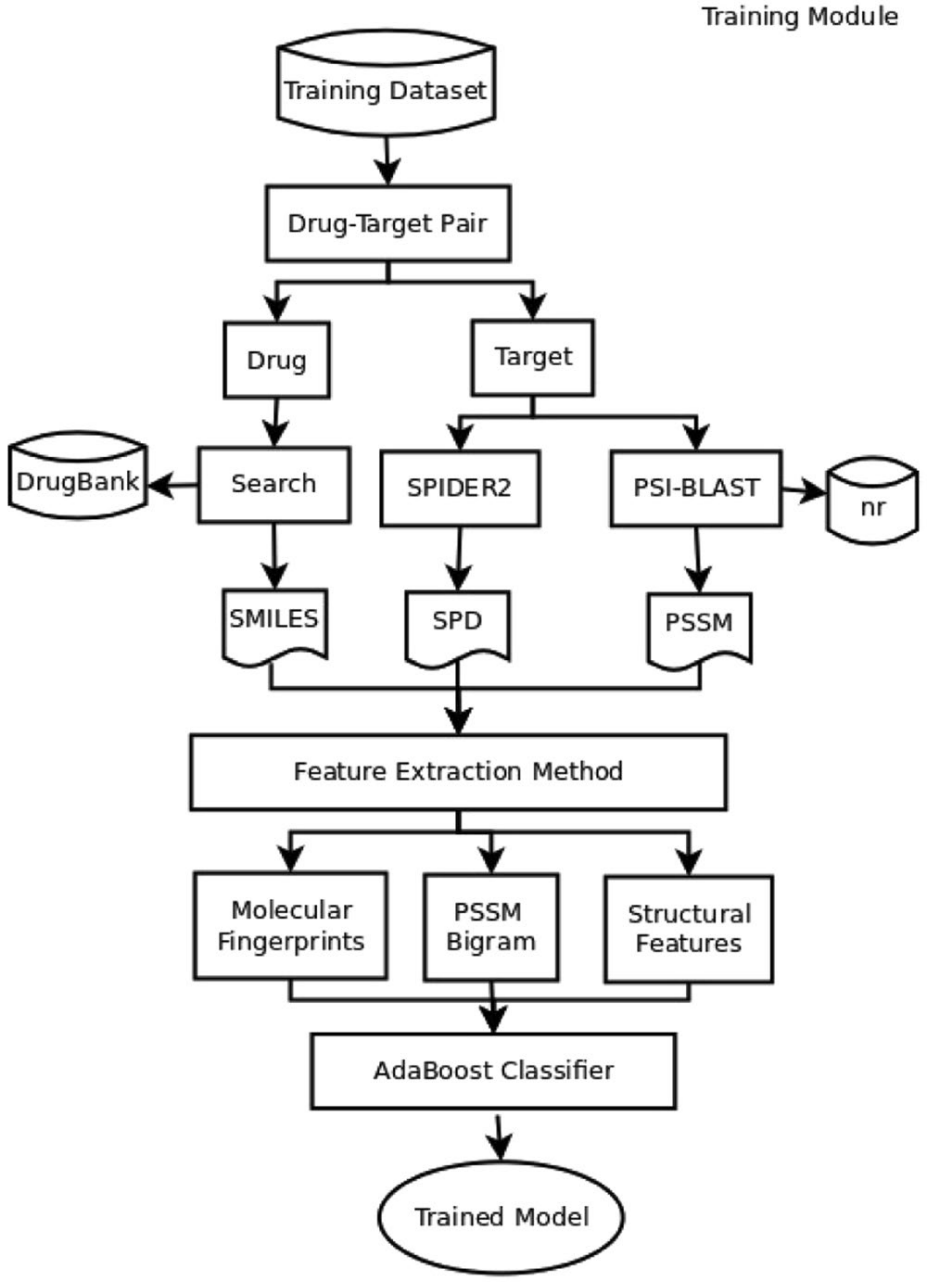
Schematic diagram of the training module of iDTI-ESBoost showing the steps of the training phase.

We have also implemented our method as shown in Fig. 5 as a separate web server. The web server is freely available to use at: http://farshidrayhan.pythonanywhere.com/iDTI-ESBoost/. The mechanism of the web-server is very simple. We also provide the pre-learned models for each of the datasets. The interface of the web server is easy to use. It requires an user first to select the target group and provide the PSSM and SPD files for the target protein. These files can be easily generated by PSI-BLAST and SPIDER2 software using their online available tools.

To specify drug, one can select from a drop down list. The drugs are pre-fetched in our system from KEGG website. After selecting the drug and specifying target files, one can click the prediction button to find the prediction for that drug-target pair. The web-server also have a simple page with easy to-use instructions. We have made all the code and programs necessary for implementation of this paper freely available at: https://github.com/farshidrayhanuiu/iDTI-ESBoost to facilitate the use of the other users.

## Materials and Methods

In this section, we provide the detail information of the benchmark datasets, feature extraction and balancing methods, classifiers and evaluation metrics used in this study. Figure 5 depicts the training module of our proposed method, iDTI-ESBoost. The training dataset of iDTI-ESBoost contains both interacting (positive) and non-interacting drug-target pairs. For each instance of drug-target pair, a drug is searched in the DrugBank database^50^ to fetch the drug chemical structure in SMILES format. Similarly, a target protein sequence is first fetched from KEGG database^51^ and then fed to SPIDER2^40,41^ and PSI-BLAST^52^ in order to receive, respectively, structural information as an SPD file and position specific scoring matrix (PSSM) based profile containing evolutionary information. A feature extraction module then uses these files to generate three types of features: drug molecular fingerprints, PSSM bigram and structural features based on the output of the secondary structure prediction software namely SPIDER2. Features generated in this phase is then fed to an AdaBoost classifier that learns the model for prediction purposes.

The prediction module is very similar to that of the training module shown in Fig. 5. For prediction, a query drug-target pair is fed to the system in a similar way to extract three types of features and then the trained and stored model is used to predict whether the given drug-target pair is interacting or non-interacting.

### Drug-target Interaction Datasets

In this paper, we have used the gold standard datasets introduced by Yamanishi *et al*. in^16^. These datasets are publicly available at: http://web.kuicr.kyoto-u.ac.jp/supp/yoshi/drugtarget/. Yamanishi *et al*. used DrugBank^53^, KEGG BRITE^54^, BRENDA^55^ and SuperTarget^56^ to extract information about drug-target interactions. They used the known drugs to four types of protein targets, namely, enzymes, ion channels, g-protein coupled receptors (GPCRs) and nuclear receptors. The number of target proteins in these groups are 664, 204, 95 and 26, respectively that interact with 445, 210, 223 and 54 drugs through 2926, 1476, 635 and 90 known interactions, respectively. A brief summary of these datasets are given in Table 9. These benchmark datasets have been used in many studies in the literature^21,22,24,27^ and are referred to as the ‘gold’ standard.

**Table 9:** Description of the gold standard datasets^16^.

Dataset	Drugs	Proteins	Positive Interactions	Imbalance Ratio
Enzyme	445	664	2926	99.98
Ion Chanel	210	204	1476	28.02
GPCR	223	95	635	32.36
Nuclear Receptor	54	26	90	14.6

### Graph Construction from the Dataset

Based on the interactions of four types of proteins with known drugs, we build positive and negative samples for each dataset using a method similar to the one used in^22^ as follows. The drug-target interaction network for each dataset is a bipartite graph, *G* = (*V*, *E*), where the set of vertices is $V=D\cup T$ such that *D* is the set of drugs and *T* is the set of targets, $D\cap T=\varnothing$ and the set of edges is *E*. Here, any edge *e* = (*d*, *t*) $\in$ *E* denotes an interaction only between a drug, *d* $\in$ *D* with a protein target, *t* $\in$ *T*. Now, for a particular graph from a dataset, all the known interactions in the graph represented by its edges are considered to be positive samples and the non-existent edges are taken as negative samples. Note that, here, non-existent edges refer to the possible valid edges only that are not there; i.e., they do not include edges among the vertices of the same partite set. Formally, a dataset is an union of positive and negative sets as follows:

1 $${\mathbb{S}}={{\mathbb{S}}}^{+}\cup {{\mathbb{S}}}^{-}$$

Here, ${{\mathbb{S}}}^{+}=\{(u,v):u\in D,v\in T,(u,v)\in E\}$, and ${{\mathbb{S}}}^{-}=\{(u,v):u\in D,v\in T,(u,v)\notin E\}$. For example, in the nuclear receptor, there are 54 drugs and 26 proteins with possible 54 × 26 = 1404 interactions. Since 90 interactions are known, these are treated to be positive and the rest 1314 as negative. The same procedure was followed for each of the datasets. As expected, the constructed datasets using this technique are imbalanced as the number of negative samples far outnumbers that of positive samples. This issue is attended to later by applying some balancing techniques. The majority class is the class denoting negative interaction. We define imbalance ratio as the number of instances in the majority class to the number of instances in the minority class. The imbalance ratio of each of the datasets used in this paper is reported in Table 9. Note that the enzymes dataset is with highest imbalance ratio near 100 and nuclear receptor dataset has got the lowest imbalance ratio.

### Feature Extraction

A dataset constructed in this way has drug-target pairs as instances. In the feature extraction phase, a drug identifier is looked up in the KEGG databased^54^ and the corresponding SMILES format is downloaded from the DrugBank database^50^. The features based on drugs are generated using this SMILES data.

Similarly, a protein target of each pair is first searched within the KEGG database^54^ to fetch the protein sequence. This protein sequence is then fed to two different software: Position Specific Iterated BLAST (PSI-BLAST)^52^ to fetch evolutionary profile based Position Specific Scoring Matrix (PSSM) and a secondary structure prediction tool called SPIDER2^40,41^ to generate SPD files that contains the structural information. Three groups of features are extracted using these three files. The details are described in the rest of this section.

#### SMILES Based Features

Several descriptors are used to represent the features or properties of drug compounds^57^. To this end, one of the most popular features is molecular fingerprints which is widely used for similarity searching^58^, clustering^59^, and classification^22^. Each drug compound is represented by 881 chemical substructures defined in PubChem database^60^. The presence (absence) of a particular substructure is encoded as 1 (0). Thus the length of this molecular fingerprint based feature is 881. We used the *rcdk* package of R^61^ to extract these molecular fingerprints based features.

#### PSSM Based Features

We used the PSSM matrix returned by the PSI-BLAST software to generate evolutionary features from the protein target sequences. Each PSSM file contains a PSSM matrix that is constructed after multiple sequence alignment using the non redundant (NR) database. The PSSM file contains a matrix *M* of dimension *L* × 20, where *L* is the length of the protein and each of the entries in this matrix, *m*_*ij*_, represents the probability of substitution of the *j*-th amino acid in the *i*-th location of the given protein sequence. We first convert this matrix *M* to a normalized matrix using a normalization technique similar to that proposed in^62,63^. The dimension of this matrix is same as the original matrix *M*. After that we generate PSSM-bigram features using the following equation:

2 $$\mathrm{PSSM} \mbox{-} \mathrm{bigram}(k,l)=\frac{1}{L}\,\sum _{i=1}^{L-1}\,{N}_{i,k}{N}_{i+\mathrm{1,}l}\,(1\le k\le 20,1\le l\le 20)$$

Bigram features for PSSM were first proposed in^64^ and subsequently used successfully in drug-target interaction prediction in^22^. Total number of features generated using this method is 400.

#### Structure Based Features

The traditional drug discovery is a lock-key problem, where the lock is the target. The structure of the target thus plays a very important role in traditional drug discovery and is at the center of the docking based software. We make a hypothesis that even if the full structure is not present for the targets, estimated structural properties still can play an important role in drug-target interaction prediction. Structural features are generated using the structural information generated and stored in SPD files by SPIDER2 software. The information generated by SPIDER2 are: accessible surface area (ASA), secondary structural (SS) motifs, torsional angles (TA) and structural probabilities (SP)^65–67^. Following features are generated using these information:

1. **Secondary Structure Composition:** This feature is the normalized count or frequency of the structural motifs present at the amino-acid residue positions. There are three types of motifs: *α*-helix (H), *β*-sheet (E) and random coil (C). SPIDER2 returns a vector *SS* of dimension *L* × 1 containing this information. Thus we can define this feature as following:
  3 $$\mathrm{SS} \mbox{-} \mathrm{Composition}(i)=\frac{1}{L}\,\sum _{j=1}^{L}\,{c}_{ij},1\le i\le 3$$
  Here, *L* is the length of the protein and
  $${c}_{ij}=\{\begin{array}{ll}1, & {\rm{if}}\,S{S}_{j}={f}_{i}\\ 0, & {\rm{else}}\end{array}$$
  Here, *SS*_*j*_ is the structural motif at position *j* of the protein sequence and *f*_*i*_ is one of the 3 different motif symbols.
2. **Accessible Surface Area Composition:** The accessible surface area composition is the normalized sum of accessible surface area defined by:
  4 $$\mathrm{ASA} \mbox{-} \mathrm{Composition}=\frac{1}{L}\,\sum _{i=1}^{L}\,ASA(i)$$
  Here ASA is the vector of accessible surface area of dimension *L* × 1 containing the values of accessible surface area for all the amino acid residues.
3. **Torsional Angles Composition:** Four different types of torsional angles: *φ*, *ψ*, *τ* and *θ* are returned by SPIDER2 for each residue. First, we convert each of them into radians from degree angles and then take sign and cosine of the angles at each residue position. Thus we get a matrix of dimension *L* × 8. We denote this matrix by *T*. Torsional angles composition is defined as:
  5 $$\mathrm{TA} \mbox{-} \mathrm{Composition}({\rm{k}})=\frac{1}{L}\,\sum _{i=1}^{L}\,{T}_{i,k}\,\mathrm{(1}\le k\le 8)$$
4. **Torsional Angles Bigram:** The Bigram for the torsional angles is similar to that of the PSSM matrix and is defined as:
  6 $$\mathrm{TA} \mbox{-} \mathrm{bigram}(k,l)=\frac{1}{L}\,\sum _{i=1}^{L-1}\,{T}_{i,k}{T}_{i+1,l}\,\mathrm{(1}\le k\le 8,1\le l\le \mathrm{8)}$$
5. **Structural Probabilities Bigram:** Structural probabilities for each position of the amino-acid residue are given in the SPD2 file as a matrix of dimension *L* × 3, which we denote by *P*. The Bigram of the structural probabilities is similar to that of PSSM matrix and is defined as:
  7 $$\mathrm{SP} \mbox{-} \mathrm{bigram}(k,l)=\frac{1}{L}\,\sum _{i=1}^{L-1}\,{P}_{i,k}{P}_{i+1,l}\,\mathrm{(1}\le k\le 3,1\le l\le \mathrm{3)}$$
6. **Torsional Angles Auto**-**Covariance:** This feature is also derived from the torsional angles and is defined as:
  8 $$\mathrm{TA} \mbox{-} \mathrm{Auto} \mbox{-} \mathrm{Covariance}(k,j)=\frac{1}{L}\,\sum _{i=1}^{L-k}\,{T}_{i,j}{T}_{i+k,j}\,\mathrm{(1}\le j\le 8,1\le k\le DF)$$
  This feature group depends on parameter DF which is the distance factor. In this study, we used DF = 10 and this value was selected for the parameter DF as it was shown as the most effective window size to extract features based on torsion angles and similar properties^40,65,67,68^.
7. **Structural Probablities Auto**-**Covariance:** This feature is also derived from the structural probabilities and is defined as:

9 $$\mathrm{SP} \mbox{-} \mathrm{Auto} \mbox{-} \mathrm{Covariance}(k,j)=\frac{1}{L}\,\sum _{i=1}^{L-k}\,{P}_{i,j}{P}_{i+k,j}\,\mathrm{(1}\le j\le 3,1\le k\le DF)$$

A brief summary of the three group of features derived from each drug-target pair is given in Table 1. Note that there are two types of features. Drug related features and target related features in four groups A, B, C and D.

### Balancing Methods

As it was specified earlier, our employed datasets are all imbalanced. Several sampling techniques in the literature have been employed to balance these data such as: random under sampling^22^, synthetic over sampling^69^, balanced random sampling (BRS)^68,70^, neighborhood cleaning rule^71^, and cluster based under sampling^72,73^. In this paper, we explore random under sampling (RUS) method as done previously for drug-target interaction prediction in^22^. We also propose a novel modified cluster based under sampling method based on^73^ as follows.

In this method, the dataset is first divided into two subsets as major class and minor class. In the major class *k*-means clustering is applied to divide the major class samples in *k* clusters while the minor class samples are kept unchanged. After that from the *k* clusters of major class samples, subsamples are chosen randomly to represent the entire major class. We denote this method as *cluster based under sampling* (*CUS*) throughout this paper. The random under sampling will be denoted as *random under sampling* (*RUS*). The pseudo-code for the CUS algorithm is given in Algorithm 1.

Our CUS algorithm depends on two parameters, namely, *k* and *h*. In our experiments, we have varied *k* for values from $5\cdots 30$ and found the the best performing value to be 23. However, more sophisticated clustering algorithms can be applied on this data. The role of the parameter *h* is to control the random under sampling of the clustered majority class samples. The details of the experimental results for selecting this hyper parameter *h* is given as supplementary material (Supplementary File 2).

**Algorithm 1:**
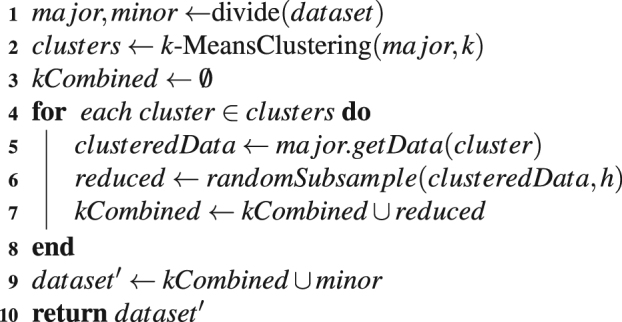
Cluster Based Under Sampling (*dataset*, *k*, *h*).

### Description of the classifier

We have selected the adaptive boosting algorithm (AdaBoost)^46^ as our classification algorithm. Adaptive boosting is a meta or ensemble classifier that uses several weak learning algorithms or weak classifiers and improves over their performance. We choose decision tree classifiers as the weak classifiers. AdaBoost is a meta-classifier of the following form:

10 $$g=\sum _{t=1}^{T}\,{\alpha }_{t}{h}_{t}(x)$$

AdaBoost iteratively adds up a weak classifier *h*_*t*_(*x*) at each iteration of the algorithm weighted by *α*_*t*_ where *α*_*t*_ is the weight achieved from the error function *ε*_*t*_ for the weak classifier *h*_*t*_(*x*) at iteration *t*. Each of these weak classifiers is chosen in a way so as to minimize the error on the training sample weighted by the distribution *D*_*t*_:

11 $${h}_{t}\in \mathop{argmin}\limits_{h\in H}\mathop{{\rm{\Pr }}}\limits_{i\sim {D}_{t}}\,[{h}_{t}(x)\ne {y}_{i}]=\mathop{argmin}\limits_{h\in H}\,\sum _{i=1}^{m}\,{D}_{t}\,{1}_{{h}_{t}(x)\ne {y}_{i}}$$

The algorithm of AdaBoost^46^ is sketched in Algorithm 2 following the notations of ^74^.

**Algorithm 2:**
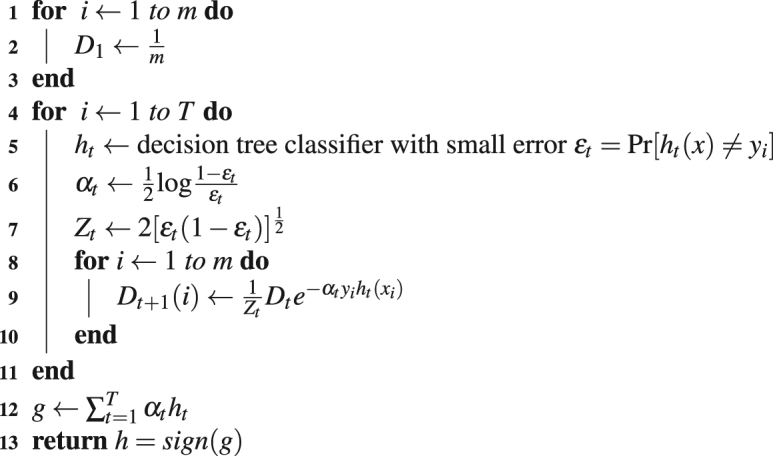
AdaBoost (*dataset* = (*X*, *Y*)).

### Performance Evaluation

A large variety of performance metrics are used in the literature to compare the performance of supervised learning methods^75^. The gold datasets that are used in the literature of drug-target interaction prediction are largely imbalanced and the number of negative samples largely outnumbers that of the positive samples. Therefore, the typical measures like accuracy does not make much sense. Moreover, the output of the classifier generating probabilistic outputs depends on the thresholds or the values predicted by it for each of the predicting classes. In such cases, thresholds or values play an important role on the sensitivity and specificity of the classifiers. Two measures that are independent of the values or thresholds set for decision making are area under curve for Receiver Operating Characteristic (auROC) and area under precision recall curve (auPR). These two measures are widely used in the literature of drug-target interaction prediction^22,24,30,76^ and thus have become standard metrics for comparison.

Lets assume, *P* is the total number of positive samples in a dataset and *N* is the total number of negative samples in a dataset. Let *TP* denote the number of true positives, *TN* denote the number of true negatives, *FN* denote the number of false negatives and *FP* denote the number of false positives predicted by a classifier. True positives (negatives) are correctly classified positive (negative) samples by the classifier. Conversely, false positives (negatives) are negative (positive) samples incorrectly predicted as positives (negatives) by the classifier. Following these notions, we can define *sensitivity* or *true positive rate* as follows:

12 $$Sensitivity=\frac{TP}{TP+FN}$$

Therefore, sensitivity is the ratio of correctly predicted positive samples to the total number of positive samples. *Precision* is defined as the positive predictive rate (PPV) as follows:

13 $$Precision=\frac{TP}{TP+FP}$$

Therefore, precision shows the percentage of positive predictions by the classifiers that are accurate. Another important measure is specificity (SPC) or true negative rate defined as follows:

14 $$SPC=\frac{TN}{TN+FP}$$

Fall-out or false positive rate (FPR) is the ration of the number of wrongly classified negative instances to the total number of negative instances defined as follows:

15 $$FPR=\frac{FP}{FP+TN}=1-SPC$$

F1 Score is a statistical score based on precision and sensitivity and defined as follows:

16 $$F1=\frac{2TP}{2TP+FP+FN}$$

All theses performance measures have values within the range [$0\cdots 1$], 0 being the worst and 1 being the best.

Another score that is often used in comparison is called defined as follows:

17 $$MCC=\frac{(TP\times TN)-(FP\times FN)}{\sqrt{(TP+FP)\,(TP+FN)\,(TN+FP)\,(TN+FN)}}$$

Value of this coefficient ranges from −1 to +1, where +1 means a perfect predictor and −1 means a total disagreement.

Receiver operating characteristic (ROC) curve plots true positive rate or sensitivity against false positive rate or (1-specificity) at various threshold values. The performance of a predictor is calculated by the area under the ROC curve (auROC). A perfect classifier have a auROC value of 1 and a random classifier have a value of 0.5. However, for imbalanced datasets like ours, area under precision recall curve (auPR) is of more significance^22^ as follows: auPR curve plots the precision rate vs the recall rate at different threshold values. This score penalizes the false positives more as compared to auROC and thus more suitable for skewed datasets. The value of auPR ranges from 0 to 1 and the higher the value is the better.

It is very important to test the methods to check and balance the bias-variance trade-off^77^. Various methods of sampling are used to measure the performance of supervised learning algorithms^78^. Among them mostly used are *k*–fold cross validation and jack knife tests. Because of the high imbalance, dimensionality and cardinality of the datasets, in most of the methods in the literature, 5-fold cross validation have been preferred and used as the sampling method^22,24,30,76^. We also use the 5-fold cross validation to test our method for the sake of fair comparison with the other state-of-the-art methods.

In the 5-fold cross validations, first the dataset is randomly split into five equal parts retaining the ratio of imbalance in each split same to the original dataset. Each time one part of the dataset is used as test and the other four are used as training data. First the balancing techniques are applied to the training data (clustered or random) and then the classifier is used to train the data into a model. The stored model is used subsequently to predict the labels for the test data. Thus all the drug-target pairs in the datasets are used in testing the classifier performance using cross-validation. The measures reported are the average of all 5-fold results.

### Data and Material availability

All the data and materials used in this paper are available at: http://farshidrayhan.pythonanywhere.com/iDTI-ESBoost/.

## Conclusion

In this paper, we have presented iDTI-ESBoost, a novel method to predict and identify drug-target interactions. iDTI-ESBoost is unique in its exploitation of structural features along with the evolutionary features to predict drug-protein interactions. It also uses a novel balancing technique and a boosting technique. We have conducted extensive experiments to test and analyze the performance of iDTI-ESBoost. On four benchmark datasets known as the gold standard data in the literature, iDTI-ESBoost outperforms the state-of-the-art methods in terms of area under Receiver Operating Characteristic (auROC) curve.

Notably, the gold standard datasets used in the literature as benchmarks to analyze the performance of the methods for drug-target interactions prediction and identification are highly imbalanced with negative samples far outnumbering the positive samples. In the literature it has been argued that area under Precision Recall (auPR) curve is the most appropriate metric for comparison for such imbalanced datasets. To this end, iDTI-ESBoost also outperforms the latest and the best-performing method in the literature to-date in terms of area under precision recall (auPR) curve. We believe that the excellent performance of iDTI-ESBoost both in terms of auROC and auPR would motivate the researchers and practitioners to use it to predict drug-target interactions. To facilitate that, iDTI-ESBoost is publicly available for use at: http://farshidrayhan.pythonanywhere.com/iDTI-ESBoost/.

In addition of target proteins, there are some types of RNA molecules so called non-coding RNAs and ncRNAs which are not translated into proteins. These RNA molecules als can make a new class of drug targets. Recently, a new database called NRDTD has been developed to collect the experimentally validated associations between drugs and ncRNAs^79^. In the future, our aim is to use NRDTD database as a gold standard dataset for predicting new associations between drugs and ncRNAs which have not been experimentally verified. By replacing features of target proteins with a set of informative features for ncRNAs, which have been published in the literature, the model presented in this study can also be used for predicting drug-ncRNA interaction prediction.

## Electronic supplementary material

Supplementary Information 1

Supplementary Information 2
