# Supplementary material for: iDTI-ESBoost: Identification of Drug Target Interaction Using Evolutionary and Structural Features with Boosting
Source: Sci Rep. 2017 Dec 18;7:17731. doi: 10.1038/s41598-017-18025-2 (PMC5735173; doi:10.1038/s41598-017-18025-2)
Supplement: Supplementary file 1 — Supplementary Information 1 [file 41598_2017_18025_MOESM1_ESM.pdf]

iDTI-ESBoost: Identification of Drug Target Interaction Using Evolutionary and Structural Features with Boosting

Farshid Rayhan[1], Sajid Ahmed[1], Swakkhar Shatabda[1,*], Dewan Md Farid[1], Zaynab Mousavian[2], Abdollah Dehzangi[3], and M Sohel Rahman[4]
[1]Department of Computuer Science and Engineering, United International University, House 80, Road 8A, Dhanmondi, Dhaka-1209, Bangladesh
[2]Department of Computer Science, School of Mathematics, Statistics, and Computer Science, University of Tehran,
Tehran, Iran
[3]Department of Computer Science, Morgan State University, Baltimore, Maryland, USA
[4]Department of Computer Science and Engineering, Bangladesh University of Engineering and Technology, Palashi, Dhaka-1000, Bangladesh
[*]corresponding swakkhar@cse.uiu.ac.bd

Supplementary Information 1

Experimental results obtained using different feature group combinations and random sampling and clustered sampling as balancing methods for different datasets

Dataset: Enzymes

Feature Group Combination	Clustered Sampling		Random Sampling	
	auROC	auPR	auROC	auPR
A	0.9493	0.58	0.953	0.54
B	0.9359	0.64	0.9034	0.45
C	0.9381	0.66	0.9356	0.50
D	0.9395	0.6	0.9287	0.50
AB	0.9353	0.59	0.9431	0.51
AC	0.9525	0.67	0.9412	0.49
AD	0.9511	0.65	0.9571	0.65
BC	0.8967	0.57	0.9445	0.58
BD	0.9254	0.67	0.9601	0.61
CD	0.9123	0.63	0.9623	0.62
ABD	0.8479	0.64	0.9572	0.60
ABC	0.9577	0.63	0.9638	0.66
ACD	0.9374	0.62	0.9599	0.67
BCD	0.9034	0.62	0.9545	0.61
ABCD	0.9598	0.68	0.9689	0.65

Dataset: GPCRs

Feature Group Combination	Clustered Sampling		Random Sampling	
	auROC	auPR	auROC	auPR
A	0.8856	0.48	0.8856	0.29
B	0.896	0.46	0.8512	0.29
C	0.8994	0.46	0.8718	0.30
D	0.9395	0.6	0.9104	0.32
AB	0.8834	0.49	0.8834	0.29
AC	0.9271	0.5	0.9056	0.35
AD	0.8855	0.47	0.9092	0.34
BC	0.9247	0.39	0.8213	0.27
BD	0.9412	0.41	0.9082	0.31
CD	0.8735	0.45	0.9023	0.29
ABD	0.9231	0.5	0.9091	0.35
ABC	0.9116	0.5	0.9116	0.35
ACD	0.8809	0.48	0.91738	0.33
BCD	0.8879	0.49	0.9096	0.34
ABCD	0.9128	0.48	0.9128	0.31

Dataset: Ion Channels

Feature Group Combination	Clustered Sampling		Random Sampling	
	auROC	auPR	auROC	auPR
A	0.8982	0.38	0.9271	0.36
B	0.8936	0.3	0.8792	0.29
C	0.8992	0.39	0.9265	0.30
D	0.8949	0.36	0.9167	0.32
AB	0.8902	0.41	0.9191	0.33
AC	0.8947	0.47	0.9098	0.37
AD	0.8984	0.44	0.9332	0.43
BC	0.841	0.38	0.9182	0.41
BD	0.8639	0.45	0.9287	0.41
CD	0.8718	0.42	0.906	0.42
ABD	0.875	0.47	0.9332	0.43
ABC	0.9021	0.45	0.9202	0.34
ACD	0.8917	0.47	0.9295	0.42
BCD	0.8721	0.39	0.9292	0.46
ABCD	0.9051	0.48	0.9369	0.43

Dataset: Nuclear Receptors

Feature Group Combination	Clustered Sampling		Random Sampling	
	auROC	auPR	auROC	auPR
A	0.8874	0.79	0.8145	0.41
B	0.8728	0.5	0.7639	0.40
C	0.9011	0.48	0.7931	0.41
D	0.8949	0.36	0.798	0.32
AB	0.8964	0.32	0.7969	0.43
AC	0.8879	0.53	0.789	0.32
AD	0.8928	0.64	0.8067	0.36
BC	0.8273	0.67	0.8282	0.19
BD	0.8734	0.71	0.8256	0.35
CD	0.8587	0.68	0.8434	0.28
ABD	0.9064	0.56	0.7942	0.30
ABC	0.8969	0.68	0.759	0.36
ACD	0.9085	0.57	0.8133	0.34
BCD	0.8943	0.72	0.8561	0.32
ABCD	0.887	0.79	0.7946	0.33

Experimental results obtained using different feature group combinations and classifiers for different datasets

Dataset: Enzymes

Feature Group Combination	Random Forest		AdaBoost		SVM	
	auROC	auPR	auROC	auPR	auROC	auPR
A	0.8698	0.42	0.953	0.54	0.8051	0.44
B	0.9409	0.37	0.9034	0.45	0.8353	0.5
C	0.9329	0.46	0.9356	0.50	0.8073	0.41
D	0.8832	0.43	0.9287	0.50	0.7984	0.51
AB	0.9315	0.39	0.9431	0.51	0.8324	0.54
AC	0.949	0.52	0.9412	0.49	0.8221	0.47
AD	0.9349	0.43	0.9571	0.65	0.7952	0.48
BD	0.8929	0.48	0.9601	0.61	0.8198	0.44
CD	0.9577	0.51	0.9623	0.62	0.8353	0.46
BC	0.8873	0.44	0.9445	0.58	0.8254	0.46
BCD	0.9058	0.44	0.9545	0.61	0.8213	0.4
ABD	0.9378	0.5	0.9572	0.60	0.7913	0.49
ABC	0.8663	0.38	0.9638	0.66	0.796	0.52
ACD	0.9484	0.5	0.9599	0.67	0.7944	0.42
ABCD	0.8973	0.46	0.9689	0.65	0.814	0.55

Dataset: GPCRs

Feature Group Combination	Random Forest		AdaBoost		SVM	
	auROC	auPR	auROC		auROC	auPR
A	0.843	0.28	0.8856	0.29	0.7914	0.03
B	0.8256	0.31	0.8512	0.29	0.8061	0.18
C	0.9056	0.31	0.8718	0.30	0.8083	0.1
D	0.8741	0.25	0.9104	0.32	0.7908	0.35
AB	0.8977	0.25	0.8834	0.29	0.8086	0.25
AC	0.8214	0.28	0.9056	0.35	0.7934	0.33
AD	0.869	0.28	0.9092	0.34	0.8071	0.34
BD	0.8976	0.28	0.9082	0.31	0.784	0.11
CD	0.8954	0.29	0.9023	0.29	0.7879	0.31
BC	0.8938	0.27	0.8213	0.27	0.7947	0.33
BCD	0.8484	0.29	0.89	0.25	0.7866	0.08
ABD	0.8227	0.29	0.9091	0.35	0.7841	0.03
ABC	0.8356	0.31	0.9116	0.35	0.7925	0.24
BCD	0.9013	0.26	0.9096	0.34	0.7849	0.16
ACD	0.8743	0.28	0.91738	0.33	0.7919	0.2
ABCD	0.895	0.28	0.9128	0.31	0.7907	0.07

Dataset: Ion Channels

Feature Group Combination	Random Forest		AdaBoost		SVM	
	auROC	auPR	auROC		auROC	auPR
A	0.8347	0.15	0.9271	0.36	0.7929	0.4
B	0.8387	0.15	0.8792	0.29	0.7405	0.27
C	0.8861	0.3	0.9265	0.30	0.819	0.26
D	0.8728	0.12	0.9167	0.32	0.7866	0.29
AB	0.891	0.26	0.9191	0.33	0.7782	0.32
AC	0.8918	0.37	0.9098	0.37	0.7593	0.23
AD	0.8947	0.14	0.9332	0.43	0.7296	0.32
BD	0.8294	0.14	0.9287	0.41	0.8151	0.3
CD	0.833	0.16	0.906	0.42	0.7513	0.27
BC	0.8274	0.38	0.9182	0.41	0.7271	0.34
BCD	0.8589	0.12	0.9292	0.46	0.7865	0.25
ABD	0.8204	0.29	0.9332	0.43	0.7696	0.21
ABC	0.8991	0.13	0.9202	0.34	0.7856	0.36
ACD	0.8104	0.34	0.9295	0.42	0.7927	0.32
ABCD	0.8868	0.16	0.9369	0.43	0.7431	0.38

Dataset: Nuclear Receptors

Feature Group Combination	Random Forest		AdaBoost		SVM	
	auROC	auPR	auROC		auROC	auPR
A	0.8433	0.79	0.8145	0.41	0.7914	0.51
B	0.8948	0.5	0.7639	0.40	0.814	0.43
C	0.8755	0.48	0.7931	0.41	0.7346	0.54
D	0.8802	0.36	0.798	0.32	0.7201	0.51
AB	0.8745	0.32	0.7969	0.43	0.7922	0.4
AC	0.8451	0.53	0.789	0.32	0.7938	0.5
AD	0.8816	0.64	0.8067	0.36	0.7348	0.49
BD	0.8449	0.71	0.8256	0.35	0.7114	0.42
CD	0.8832	0.68	0.8434	0.28	0.7331	0.4
BC	0.8716	0.67	0.8282	0.19	0.7872	0.49
BCD	0.8813	0.72	0.8561	0.32	0.7988	0.5
ABD	0.8518	0.56	0.7942	0.30	0.8095	0.5
ABC	0.8819	0.68	0.759	0.36	0.7956	0.46
BCD	0.8793	0.7	0.8145	0.35	0.7433	0.45
ACD	0.899	0.57	0.8133	0.34	0.7532	0.5
ABCD	0.8973	0.79	0.7946	0.33	0.7118	0.44
