# Supplementary material for: iDTI-ESBoost: Identification of Drug Target Interaction Using Evolutionary and Structural Features with Boosting
Source: Sci Rep. 2017 Dec 18;7:17731. doi: 10.1038/s41598-017-18025-2 (PMC5735173; doi:10.1038/s41598-017-18025-2)
Supplement: Supplementary file 2 — Supplementary Information 2 [file 41598_2017_18025_MOESM2_ESM.pdf]

iDTI-ESBoost: Identification of Drug Target Interaction Using Evolutionary and Structural Features with Boosting

Farshid Rayhan[1], Sajid Ahmed[1], Swakkhar Shatabda[1,*], Dewan Md Farid[1], Zaynab Mousavian[2], Abdollah Dehzangi[3], and M Sohel Rahman[4]
[1]Department of Computuer Science and Engineering, United International University, House 80, Road 8A, Dhanmondi, Dhaka-1209, Bangladesh
[2]Department of Computer Science, School of Mathematics, Statistics, and Computer Science, University of Tehran,
Tehran, Iran
[3]Department of Computer Science, Morgan State University, Baltimore, Maryland, USA
[4]Department of Computer Science and Engineering, Bangladesh University of Engineering and Technology, Palashi, Dhaka-1000, Bangladesh
[*]corresponding swakkhar@cse.uiu.ac.bd

Supplementary Information 2

Experimental results obtained using different values of the hyper parameter h of clustered sampling as balancing methods for different datasets

Dataset: Enzymes

Value of h	auROC	AUPR
25%	0.9478	0.66
50%	0.9598	0.68
75%	0.9398	0.65

Dataset: GPCRs

Value of h	auROC	AUPR
25%	0.9187	0.46
50%	0.9322	0.48
75%	0.9299	0.45

Dataset: Ion Channels

Value of h	auROC	AUPR
25%	0.9398	0.41
50%	0.9369	0.48
75%	0.9123	0.42

Dataset: Nuclear Receptors

Value of h	auROC	AUPR
25%	0.9012	0.71
50%	0.9285	0.79
75%	0.8942	0.74
